# Fistulotomy for superficial or minimal sphincter-involving fistulae in perianal Crohn’s disease: do they heal?

**DOI:** 10.1007/s10151-026-03351-3

**Published:** 2026-05-13

**Authors:** Oscar Hernandez Dominguez, Janell Holloway, Anuradha Bhama, Benjamin L. Cohen, Leonardo Duraes, Arielle Kanters, Olga Lavryk, Jeremy Lipman, David Liska, Stefan D. Holubar

**Affiliations:** Digestive Diseases Institute, Cleveland Clinic Main Campus, Cleveland Clinic Foundation, Cleveland Clinic Lerner College of Medicine and Case Western Reserve University, 9500 Euclid Ave, Cleveland, OH A3044122 USA

**Keywords:** Fistulotomy, Fecal incontinence, Perianal Crohn’s disease, Inflammatory bowel disease, Fistula-in-ano

## Abstract

**Background:**

Fistulotomy is highly effective (> 90%) for cryptoglandular fistula-on-ano, but fistulotomy in perianal Crohn’s disease (pCD) is limited due to increased risk of recurrent fistulae, diarrhea, and fecal incontinence. We hypothesized that superficial fistulotomy resulted in wound healing in most patients.

**Method:**

We conducted a single-center retrospective review of adult patients with pCD who underwent fistulotomy from 1999 to 2022. Baseline characteristics, pCD characteristics, and short- and long-term surgical and functional outcomes were reported. Matched-pair Wilcoxon signed-rank test was used to compare continuous data.

**Results:**

A total of 43 adult patients with pCD underwent fistulotomy and were included: 29 (67.4%) were male, with an age of 34 (28–42) years and a follow-up time of 4.3 years. Prior interventions included draining setons (48.8%) and partial fistulotomy (17.4%); 25.6% had no prior surgery. Fistulotomies were subcutaneous (65.1%), low transsphincteric (16.3%), intersphincteric (4.7%), and unspecified (14%). Short-term complications included pain (20.9%), bleeding (4.7%), and seepage (2.3% each), and 58.1% of the patients had no complications. Long-term complications included bleeding, keyhole deformity, nonhealing wounds, and anal stricture (2.3% each), and 60.5% did not experience long-term complications. At the last follow-up, 41 (95.3%) patients had complete healing of the fistulotomy site.

**Conclusions:**

Fistulotomy was safe in select patients with fistulizing pCD and superficial fistulas with little or no sphincter involvement. We observed that fistulotomy was associated with wound healing, decreased drainage, and social restrictions in most patients, suggesting that this is a viable and safe option for this at-risk group.

**Supplementary Information:**

The online version contains supplementary material available at 10.1007/s10151-026-03351-3.

## Introduction

Crohn’s disease (CD) is a persistent inflammatory condition affecting the gastrointestinal tract, marked by inflammation that may penetrate the intestinal mucosa and anal canal, resulting in complications such as perianal abscesses and fistulae. Many patients with CD have a fistulizing phenotype, and the rate of fistulizing perianal CD (pCD) varies between 17% and 34% among individuals with CD [1, 2]. This disease can have an overall detrimental impact on patients’ quality of life (QoL) owing to symptoms such as chronic local pain, discharge, and fecal incontinence.

Clear guidelines on fistulizing pCD therapy in clinical practice have been fragmented until recently, and the work of the pCD Treatment Optimization and Classification (TOpCLass) Consortium represents a major advance toward synchronizing clinical practice for fistulizing pCD [3, 4]. Typically, the first-line medical treatment of pCD includes a combination of antibiotics, biologics, and immunomodulator therapies to alleviate inflammation and facilitate the healing of perianal fistulas [5]. However, medical management alone can lead to a loss of treatment response in up to half of the patients and a recurrence of fistulas [6]. Although there have been significant developments in the medical treatment of pCD, including stem cell therapy, surgical intervention remains a crucial component of the therapeutic strategy, with most individuals with pCD requiring perianal surgery [2]. Surgical management relies on surgical drainage of abscesses, control of fistulas with seton drains, and subsequent fistula repair after optimization of medical management. It is no longer advisable to rely solely on chronic seton treatment for pCD, therefore other surgical approaches have become increasingly important [7].

**Fig. 1:**
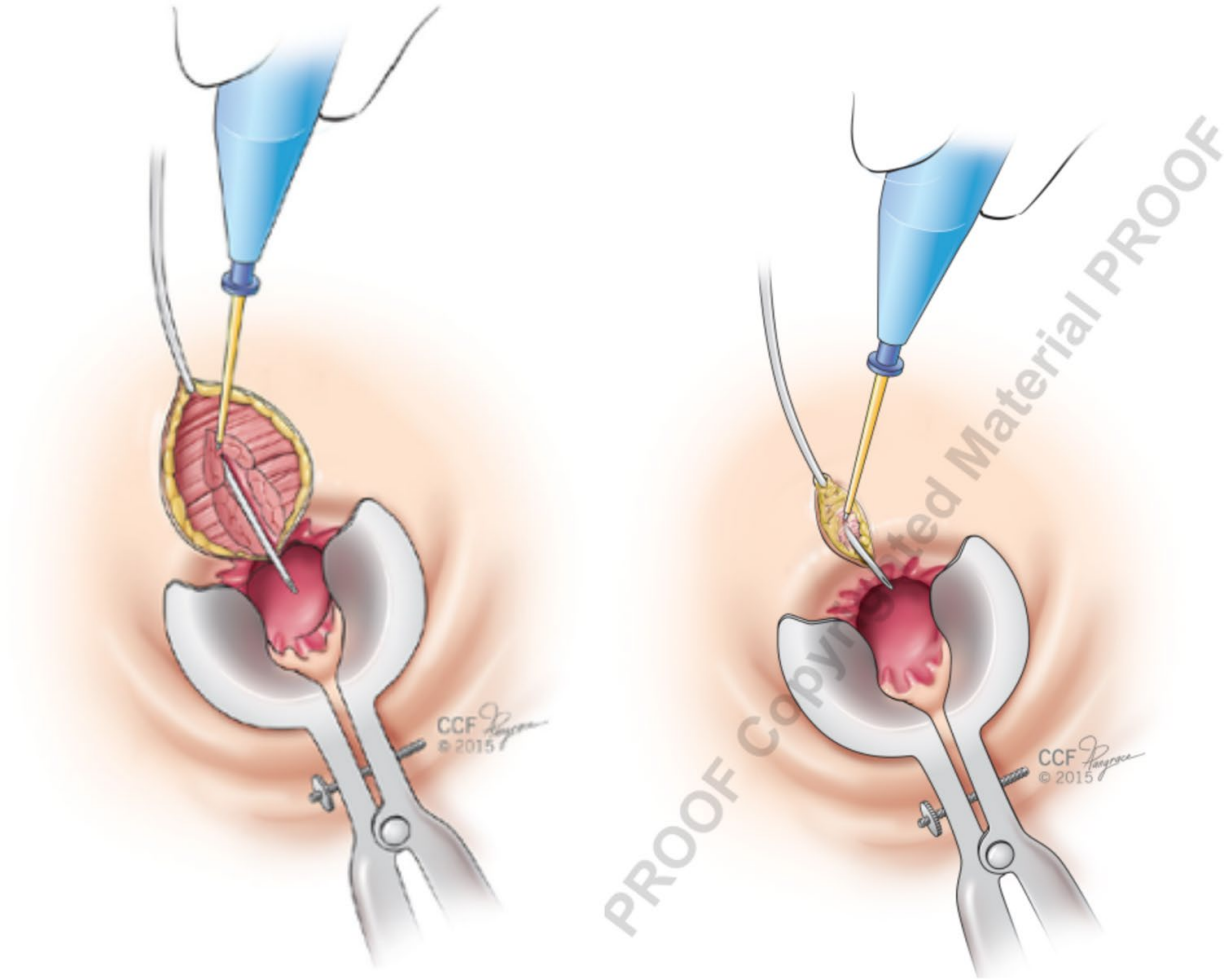
Fistulotomy. The left panel depicts cauterization of anal sphincter muscle fibers for transphincteric and intersphincteric fistulas. The right panel depicts superficial fistulotomy of subcutaneous fistula

Fistulotomy (Fig. 1) is an effective treatment option for cryptoglandular-related perianal fistulas; however, special consideration must be given to patients with fistulizing pCD because of the risk of incontinence in those prone to recurrent fistulae and chronic diarrhea related to intestinal inflammation and loss of small bowel length. Fecal incontinence, which negatively impacts health-related QoL, has been reported in 59% of patients with pCD [8, 9]. Additionally, aggressive surgical treatment for pCD may increase the risk of incontinence, as reported in up to 23% of patients undergoing anorectal surgery for pCD [9]. Other concerns with surgery include wound healing problems and anal stricture. Patients with uncontrolled pCD have a high risk of fistula recurrence, raising concerns that they may require repeated surgical procedures that compromise the anal sphincter or result in anal stenosis. However, more recent studies have reported that patients with pCD can have positive outcomes, such as high healing rates, low recurrence rates, and low risk of incontinence after fistulotomy. Consequently, specialists have advocated reconsideration of avoiding these procedures in selected patients with pCD [10–14]. Nevertheless, many surgeons continue to avoid fistulotomies in patients with pCD because of the lack of long-term data. Therefore, the long-term effects of fistulotomy on the functionality of patients with pCD should be explored further.

Therefore, we retrospectively analyzed consecutive adult patients who underwent fistulotomy for fistulizing pCD and assessed short- and long-term outcomes, such as functionality and incontinence, to determine whether fistulotomy may be safely considered a viable option for select patients with fistulizing pCD. We hypothesized superficial fistulotomy resulted in wound healing in most patients.

## Methods

We performed an Institutional Review Board (IRB)-approved retrospective review of consecutive adult patients with pCD who underwent fistulotomy between January 1999 and November 2022 at a quaternary center with a dedicated inflammatory bowel disease (IBD) division. Patients were identified using International Classification of Diseases (ICD-9 and -10) codes for CD (555.0 and K-50, respectively) and common procedural terminology (CPT) codes for fistulotomy (46,270, 46,275, 46,280, and 46,285) to identify the index fistulotomy. Patients with anovaginal or rectovaginal fistulae, ulcerative colitis (UC), or indeterminate colitis were excluded. Patients who were first diagnosed with UC and later diagnosed with CD were included. Patients with ileal pouch-anal anastomosis (IPAA) were excluded.

A retrospective chart review was performed to review clinical notes and collect variables, including patient demographics and baseline characteristics, including details regarding the date of CD diagnosis, pCD characteristics, prior treatments and operations, fistula location and characteristics, short-term (< 30 days) and long-term (> 30 days) outcomes of fistulotomy, pre- and post-fistulotomy use of immunomodulatory medications, reoperations, and functional outcomes (including incontinence) at the most recent follow-up. If a second fistulotomy was performed, its location and complications were also recorded.

**Fig. 2:**
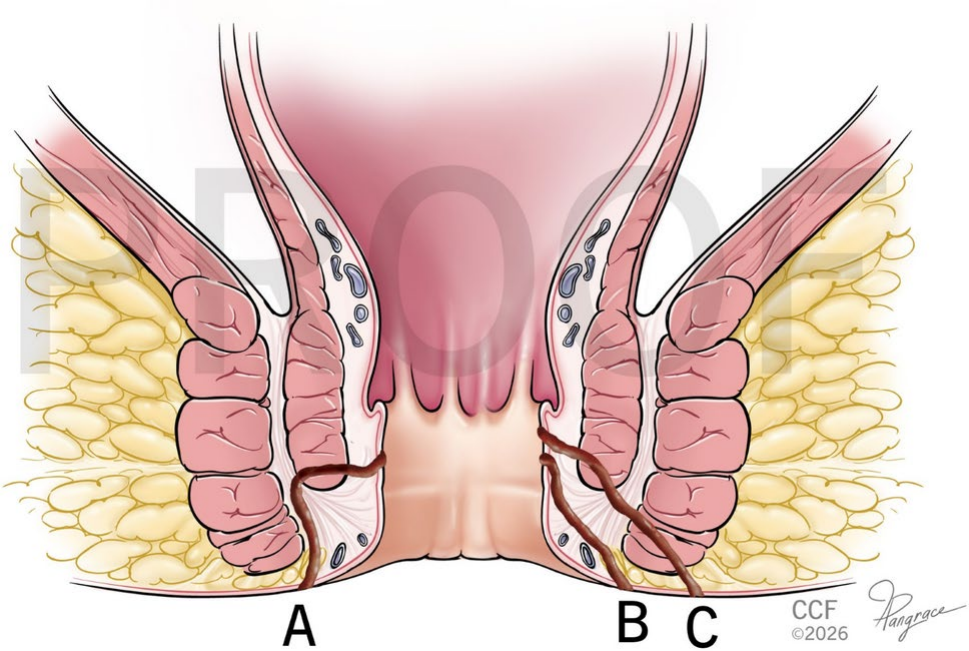
Modified Parks classification of perianal fistulae. **A** intersphincteric fistula, **B** superficial/subcutaneous fistula, **C** low-transphincteric fistula

Fistulas were categorized according to a modified Parks classification (Fig. 2) on the basis of surgical findings and imaging; the modification includes superficial fistulae that do not include any sphincter muscle [15]. Imaging of the fistula included examination under anesthesia (EUA) prior to fistulotomy, magnetic resonance imaging (MRI), endoscopy, and computed tomography (CT). Fistulotomy was broadly categorized on the basis of the operative records of the operating surgeon and the length of the anal sphincter below the internal opening of the fistula. It was categorized as follows: “superficial” if no sphincter muscle was involved, “low fistulotomy” if < 10% of muscles were involved, and “high fistulotomy” if > 10% of the muscles were involved. Patients who underwent partial fistulotomy (i.e., those in whom the external openings [EO] were not connected to the internal opening [IO]) or those without an IO in the anal canal were excluded.

The onset of new symptoms of incontinence to flatus, liquid, or solid stool in the postoperative period after fistulotomy in the absence of diarrhea was used as a marker of functional impairment caused by fistulotomy. Fecal incontinence was extracted from clinic/medical visit notes and chart reviews. Clinical healing of the pCD fistula on the date of the last clinical encounter was defined as a clinical note reporting a healed fistula, patient reported healing, and/or a normal perianal examination. Medications were classified as immunomodulators (azathioprine and 6-mercaptopurine), budesonide, or biologic therapies, such as tumor necrosis factor inhibitors (TNFi), including infliximab, adalimumab, certolizumab pegol, and other biologics, including vedolizumab and ustekinumab.

Descriptive statistics were computed for all variables, including the mean (standard deviation) or median (interquartile range) for continuous factors and frequency (%) for categorical variables. A matched-pair Wilcoxon signed-rank test was used to compare continuous data. Estimated median group differences were reported with two- and one-sided *p*-values, with *p* < 0.05 considered significant. All analyses were performed using R version 3.1.2 (www.R-project.org).

## Results

A total of 43 adult patients with pCD met the study’s inclusion criteria. Demographics and baseline characteristics of the study participants are presented in Table 1; 29 patients were men (67.4%), and median age at the time of fistulotomy was 34 (28–42) years. In total, 8 patients (18.6%) had a family history of CD, and 25 (58.14%) had additional pCD stigmata, including anal fissures (20.9%), skin tags (18.6%), anal ulcers (14%), and anal strictures (2.3%). Patients were diagnosed with CD a median of 7 (IQR 1–18.5) years before fistulotomy. The predominant fistula symptoms were mucopurulent drainage (74.4%), painful bowel movements (72.1%), and bloody drainage (22%). At the time of fistulotomy, 21 (48.8%) patients were receiving biologics, including infliximab (11.6%), ustekinumab (11.6%), adalimumab (9.3%), and vedolizumab (9.3%). Nonbiologic monotherapy included mesalamine (7%), budesonide (4.7%), methotrexate (2.3%), and azathioprine (2.3%). A total of 15 patients (34.9%) were not receiving medical treatment for CD at the time of fistulotomy. The medical management of pCD before and after fistulotomy is summarized in Table 2. Patients remained on the same medical management (48.8%), switched agents (16.3%), discontinued all therapies (14%), or initiated therapy (20.9%).

**Table 1:** Patient demographics and baseline characteristics (*N* = 43)

Variable	*N* = 43 (100%)
Males	29 (67.4%)
BMI, kg/m^2^	24.3 (21.7—30.1)
Age at fistulotomy, years	34 (28–42)
CD diagnosis to pCD diagnosis, years	1 (0–10.5)
CD diagnosis to fistulotomy, years	7 (1–18.5)
Diagnosis changed from UC to CD	3 (7.0%)
Comorbidities	
None	16 (37.2%)
Obesity	10 (23.3%)
Depression/anxiety	9 (20.9%)
Smoker	5 (11.6%)
Hypertension	5 (11.6%)
Diabetes mellitus	3 (7.0%)
Venous thromboembolism	2 (4.7%)
Cancer, any	2 (4.7%)
Coronary artery disease	1 (2.3%)
Congestive heart failure	1 (2.3%)
Chronic obstructive pulmonary disease	1 (2.3%)
Family history of IBD	
Crohn’s disease	8 (18.6%)
Ulcerative colitis	1 (2.3%)
Other pCD stigmata	
Anal fissure	9 (20.9%)
Skin tag	8 (18.6%)
Anal ulcer	6 (14.0%)
Anal stricture	2 (2.3%)
Preoperative fistula symptoms	
Mucopurulent drainage	32 (74.4%)
Pain with bowel movements	31 (72.1%)
Bloody drainage	10 (23.3%)
Skin dermatitis/irritation	7 (16.3%)
Fever	3 (7.0%)
Stool drainage	2 (4.7%)
Surgical treatment of prior pCD	
Abscess incision and drainage	28 (65.1%)
Loose seton	22 (51.2%)
Partial fistulotomy	11 (25.6%)
Cutting seton	2 (4.7%)
Fistula plug	1 (2.3%)
LIFT	1 (2.3%)
ERAF	1 (2.3%)
Fibrin glue	1 (2.3%)
No prior surgery	12 (27.9%)

Data are presented as frequency (proportion) or median (interquartile range). BMI, body mass index; CD, Crohn’s disease; UC, ulcerative colitis; pCD, perianal CD; ERAF, endorectal advancement flap; LIFT, ligation of intersphincteric fistula

**Table 2:** Medical therapy for pCD at the time of and after fistulotomy (*N* = 43)

Variable	Before	After
Biologics		
No biologic	22 (51.2%)	15 (34.9%)
Any	21 (48.8%)	28 (65.1%)
Infliximab	5 (11.6%)	5 (11.6%)
Ustekinumab	5 (11.6%)	7 (16.3%)
Adalimumab	4 (9.3%)	6 (14.0%)
Vedolizumab	4 (9.3%)	3 (7.0%)
Upadacitinib	0 (0.0%)	1 (2.3%)
Nonbiologic medical therapy		
Mesalamine	3 (7.0%)	2 (4.7%)
Budesonide	2 (4.7%)	0 (0.0%)
Methotrexate	1 (2.3%)	0 (0.0%)
Azathioprine	1 (2.3%)	1 (2.3%)
Combined medical therapy		
Infliximab + 6-MP	1 (2.3%)	0 (0.0%)
Adalimumab + 6-MP	1 (2.3%)	0 (0.0%)
Infliximab + sulfasalazine	1 (2.3%)	0 (0.0%)
Infliximab + azathioprine	0 (0.0%)	1 (2.3%)
Infliximab + mesalamine	0 (0.0%)	2 (4.7%)
Same medical management	–	21 (48.8%)
Switched agents	–	7 (16.3%)
Discontinued all therapy	–	6 (14.0%)
Initiated therapy	–	9 (20.9%)

Data are presented as frequency (proportion). 6-MP, 6-mercaptopurine

The fistula characteristics and fistulotomy details of the treated fistulas are presented in Table 3. Prior to fistulotomy, surgical treatment of pCD predominantly included incision and drainage (65.1%), loose draining seton placement (51.2%), and partial fistulotomy (25.6%). A total of 12 patients (27.9%) had no history of prior surgery. Prior surgical treatment of fistulas that underwent fistulotomy included seton placement (48.8%), incision and drainage (46.5%), partial fistulotomy (*n* = 4, 17.4%), and no surgical treatment (*n* = 11, 25.6). The duration of the draining seton placement was 5.4 (IQR 2.1–14.8) months before fistulotomy. Fistula evaluation predominantly included EUA (34.8%) and MRI (30.2%). A total of 18 patients (41.9%) had multiple fistulas at the time of fistulotomy, and 3 of those underwent multiple (two max) superficial fistulotomies in the same setting. Fistulotomies were subcutaneous (65.1%), low transsphincteric (16.3%), low intersphincteric (4.7%), or not specified in the operative report (*n* = 6, 14%).

**Table 3:** Index fistula evaluation and treatment details (*N* = 43)

Variable	*N* = 43 (100%)
Fistula treatment prior to fistulotomy, same fistula	
Medical	28 (65.1%)
Seton	21 (48.8%)
Incision and drainage	20 (46.5%)
Partial fistulotomy	4 (17.4%)
No treatment	11 (25.6%)
Seton to fistulotomy, months (*n* = 21)	5.4 (2.1; 14.8)
Location of seton placement (*n* = 21)	
Transsphincteric	6 (14.0%)
Intersphincteric	4 (17.4%)
Submuscular, not specified	1 (2.3%)
Not specified	10 (23.3%)
Work-up of fistula	
Exam under anesthesia	15 (34.8%)
Magnetic resonance imaging	13 (30.2%)
Computed tomography	4 (17.4%)
Colonoscopy	2 (4.7%)
Fistula internal opening	
Below to dentate	19 (44.2%)
Anal verge	6 (14.0%)
Dentate	5 (11.6%)
Not specified	13 (30.2%)
Fistulotomy location Posterolateral Anterolateral Posterior midline Anterior midline	15 (34.8%) 14 (32.5%) 12 (27.9%) 2 (4.7%)
Multiple fistulas	18 (41.9%)
Fistulotomy type	
Superficial/subcutaneous	28 (65.1%)
Low fistulotomy (< 10% muscle fibers)	15 (34.8%)
Any external anal sphincter divided	7 (16.3%)
Submuscular, sphincter not specified	6 (14.0%)
Internal anal sphincter divided	2 (4.7%)
Fistulotomy to last follow-up, years	4.3 (2.0–5.9)

Data are presented as frequency (proportion) or median (interquartile range)

Median follow-up time of the cohort was 4.3 (2–5.6) years after fistulotomy. Most patients (*n* = 25, 58.1%) did not experience any short-term complications. Short-term complications potentially related to fistulotomy included pain that required additional medical evaluation, such as clinic visits, emergency department visits, and pain prescriptions (*n* = 9, 20.9%). Additional short-term complications included bleeding (*n* = 2, 4.7%) and seepage (*n* = 1, 2.3%). Long-term complications included nonhealing wounds (*n* = 1, 2.3%) and anal strictures (*n* = 1, 2.3%). A total of 26 patients (60.5%) did not experience any long-term complications. Three patients (7.0%) required at least one additional surgical intervention at the sites of the initial fistula or fistulotomy: two patients underwent curettage of the wound bed incision, while one patient needed an incision and drainage followed by a subsequent fistulotomy. One patient (2.3%) continued to have blood/mucopurulent drainage, and one (2.3%) had persistent skin irritation/local dermatitis at the site of the fistulotomy. Almost all patients (*n* = 41, 95.3%) showed clinical healing of the fistula at last follow-up (Table 4). Almost half of the patients continued to have symptoms of pCD, with the majority requiring at least another pCD-related surgery.

**Table 4:** Short-term (< 30-day) and long-term (> 30-day) outcomes after fistulotomy, *N* = 43

Fistulotomy complications	Short-term *N* = 43 (%)	Long-term *N* = 43 (%)
None	25 (58.1%)	26 (60.5%)
Any	18 (41.9%)	17 (39.5%)
Bleeding	2 (4.7%)	1 (2.3%)
Keyhole deformity	0 (0.0%)	1 (2.3%)
Nonhealing wounds	0 (0.0%)	1 (2.3%)
Pain	9 (20.9%)	5 (11.6%)
Mucopurulent drainage	6 (14.0%)	5 (11.6%)
Seepage	1 (2.3%)	0 (0.0%)
Fecal diversion	0 (0.0%)	0 (0.0%)
Stricture	0 (0.0%)	1 (2.3%)
Recurrence of abscess	0 (0.0%)	3 (7.0%)
Incontinence	0 (0.0%)	0 (0.0%)
Reoperation on same fistula		
Once	0 (0.0%)	2 (4.7%)
Multiple	0 (0.0%)	1 (2.3%)
Fistulotomy healing at last follow-up		41 (95.3%)
Symptoms: Bloody drainage	–	1 (2.3%)
Symptoms: Skin irritation/dermatitis	–	1 (2.3%)
Other pCD related surgeries post-fistulotomy		
1	–	7 (16.3%)
2	–	6 (14.0%)
3 +	–	8 (18.6%)
pCD and/or treatment complication (excluding fistulotomy)		
Nonhealing wounds	–	2 (4.7%)
Fecal diversion	–	5 (11.6%)
Stricture	–	1 (2.3%)
Incontinence	–	1 (2.3%)

Data are presented as frequency (proportion) or median (IQR)

Functional outcomes before and after fistulotomy were not significantly different in terms of nocturnal seepage (2 versus 0, *p* = 0.08), nocturnal incontinence (1 versus 1, *p* = 0.5), and fecal urgency (3 versus 1, *p* = 0.08). Additionally, anal strictures due to fistulotomy were not significantly different between the groups (2 versus 2, *p* = 0.92). Fewer patients reported daytime leakage without pad use (3 versus 0, *p* = 0.04) and social restrictions (4 versus 1, *p* = 0.04) after fistulotomy than before fistulotomy. After fistulotomy, compared with before fistulotomy, more patients reported no functional complaints: 86% versus 74%, *p* = 0.05 (Table 5).

**Table 5:** Functional outcomes and anal stricture before and after index fistulotomy (*N* = 43)

Variable	Before fistulotomy	After fistulotomy	*p*-Value
No complaints	32 (74.4%)	37 (86.0%)	0.05
Day fecal incontinence	0 (0.0%)	0 (0.0%)	–
Night leakage	2 (4.7%)	0 (0.0%)	0.08
Night incontinence	1 (2.3%)	1 (2.3%)	0.5
Day leakage with pad use	1 (2.3%)	1 (2.3%)	0.5
Day leakage without pad use	3 (7.0%)	0 (0.0%)	0.04
Fecal urgency	3 (7.0%)	1 (2.3%)	0.08
Social restrictions due to function	4 (9.3%)	1 (2.3%)	0.04
Anal stricture	2 (4.7%)	2 (4.7%)	0.92

Data are presented as frequency (proportion)

None of the patients in this series required proctectomy due to adverse outcomes related to fistulotomy. However, almost half (*n* = 21, 48.8%) of the patients underwent at least one subsequent surgical procedure for pCD after the index surgery. Table 5 presents that a minority of patients (*n* = 5, 11.6%) with severe pCD required subsequent fecal diversion due to the progression of pCD. Other long-term adverse outcomes included wound deformities (*n* = 3, 7%), strictures (*n* = 1, 2.3%), and incontinence (*n* = 1, 2.3%). Nine (20.9%) patients underwent a second superficial fistulotomy, and three (7%) underwent a second low fistulotomy. The median time from the index to the second fistulotomy was 9.2 months. The median follow-up interval after the second fistulotomy was 23.1 (7.2, 38.2) months. Complications from the second fistulotomy included nonhealing wounds (*n* = 1, 8.3%), pain (*n* = 1, 8.3%), and mucopurulent drainage (*n* = 1, 8.3%). No patient reported incontinence related to the second fistulotomy, and nine patients (75%) were asymptomatic (Supplementary Table).

## Discussion

Fistulizing pCD is a challenging condition to treat owing to the recurrent and chronic nature of the disease, which can significantly diminish a patient’s QoL. Despite advances in medical management, including the use of biologics, small molecules, and stem cell therapy, surgical intervention remains an important adjunctive treatment option. However, fistulotomy has traditionally been avoided in patients with pCD because of concerns regarding the risk of incontinence in this at-risk patient population. This study demonstrated that primary superficial and low sphincter fistulotomies in select patients with pCD have very low rates of short- and long-term complications, particularly those resulting in permanent sphincter injury, and resulted in improved clinical outcomes and wound healing in 95% of the patients.

In our study, we also observed that the functional outcomes after fistulotomy were either similar or improved. No reported worsening of fecal incontinence occurred after the procedure. Although the cohort was small, patients who underwent multiple fistulotomies had similar safety and function-preserving outcomes. This study suggests that fistulotomies in patients with pCD may be safely considered for anal fistulas that are superficial or involve minimal sphincter muscle involvement.

Fistulotomy for pCD remains a contentious treatment strategy because of the risk of fecal incontinence and decreased healing time. However, recent studies have indicated that fistulotomy may be effective in certain cases [14]. The Crohn’s & Colitis Foundation of America, European Crohn’s and Colitis Organization (ECCO), and American Society of Colon and Rectal Surgeons (ASCRS) suggest that fistulotomy can be a safe and effective treatment for selected low-lying fistulas in pCD [16–18]. Our study corroborates these recommendations, as the fistulotomies in our series demonstrated low short- and long-term complications, no negative long-term functional complications, and high healing rates.

Sangwan et al. evaluated 35 patients with CD who underwent fistulotomy, and none experienced fecal incontinence in the immediate postoperative period as a direct result of the operation [12]. Our study corroborates their findings; however, we also discovered that incontinence and other functional outcomes did not develop at 4.3-year follow-up. Additionally, our study found that fistulotomy was effective, with a 95% clinical fistula healing rate. Our findings agree with recent literature showing fistulotomy for superficial or simple fistulas without proctitis tends to heal well and more effectively than with medical management and conservative seton surgery alone [6, 7, 13, 14, 18–20].

The successful treatment rates achieved in this series may be attributed to the patients receiving a personalized multimodal treatment strategy that included medical therapy to achieve fistula healing and low recurrence. In this study, medical treatment, including biological therapy, was tailored to the individual patient’s pCD, which may have improved fistula healing rates. Approximately 30% of the patients in our study were on TNFi therapy, and 20% were on other biologics. Recent research has suggested that continuing biological therapy may be beneficial for disease control and facilitate perineal wound healing without increasing postoperative infections [21–24]. Studies have suggested that the class of biologic medications may be switched to improve fistula healing and reduce pCD recurrence after fistulotomy [25]. In our study, approximately 20% of patients underwent a change in their biologic medication after fistulotomy. Further studies are necessary to determine whether changing the class of biologic medication is as effective in pCD as it is in colonic diseases.

MRI is another potential strategy for individualizing pCD treatment. A retrospective analysis evaluated the predictive value of the degree of fibrosis and disease activity (MAGNIFI-CD index) and showed that post-treatment MRI grading MAGNIFI-CD index was accurate in predicting long-term clinical closure and seems valuable in follow-up of pCD [26]. Recent studies have suggested that MRI should be standardized to measure pCD outcomes in adult and pediatric patients [27, 28]. In our series, MRI was not standardized and was performed in less than a third of patients and may be related to the ease of access for EUA at our center; however, improving MRI utilization for patients with pCD may be an area of further improvement, especially as MRI indices become standardized and comparable between studies [29]. Our results align with recent evidence indicating that fistulotomy requires the coordinated involvement of medical and surgical disciplines to provide a thorough assessment and treatment plan tailored to the individual scenario and patient goals [30, 31].

A patient’s history of prior fistulotomy is a critical factor in selecting a suitable treatment for pCD fistulas. Repeated procedures to treat recurrent fistulas in patients with pCD may ultimately harm the sphincter and lead to incontinence. Papaconstantinou et al. reported that patients undergoing fistulotomy should be carefully selected, with a history of previous fistulotomy being a significant consideration to avoid further treatment [19]. However, there is a paucity of literature analyzing the outcomes of patients with pCD after multiple fistulotomies. In our series, 12 patients underwent a second fistulotomy for superficial or low fistulas. Complications related to sphincter disturbance were few, with only one patient reporting wound healing disturbances and no patient with incontinence related to the fistulotomy. Although there were no serious complications of a second fistulotomy in our series to justify the absolute avoidance of the procedure, as previously suggested, it must be noted that our series was small, and the follow-up time was only 23 months. However, these patients were carefully selected at an IBD referral center and underwent multidisciplinary evaluation and treatment.

The strengths of this study include its sample size, which is larger than that reported to date, the duration of follow-up, and the functional outcomes. However, our study has some limitations. First, the sample was limited to patients from a single IBD referral center, which may have introduced selection bias in patients referred to the institution. Surgeons selected patients who were deemed good candidates for fistulotomy on the basis of clinical characteristics and acumen. Second, fistula healing was defined as a documented physical examination of a healed fistula or normal perianal examination. Given the retrospective nature of this study, validated scales or MRI scans were not used to confirm the healing rate, which may have demonstrated the persistence or recurrence of the fistula. Additionally, the study lacked a validated scale for incontinence or patient-reported outcome measures for functional measures, and this information was extracted from outpatient visits. We attempted to capture functional changes on the basis of gastrointestinal (GI) outpatient visits that routinely collect functional outcomes in patients with IBD as part of their clinic template. Although this is one of the largest series of fistulotomies in patients with pCD, the number of patients remains small, especially those who underwent preoperative proctitis or a second fistulotomy. This limitation restricted our ability to draw definitive conclusions regarding the outcomes of various medical regimens for treating preoperative proctitis. Additionally, due to the retrospective nature of the study, specific descriptions of the preoperative status of the anal canal were limited. Finally, we did not compare the outcomes between patients with cryptoglandular-related fistulas. Despite these limitations, our study contributes to the ongoing debate regarding the management of pCD with fistulotomy.

In conclusion, we observed that patients with superficial or low pCD fistulae and without proctitis may safely undergo careful fistulotomy for superficial or minimal sphincter-involving anal fistulas. Fistulotomy was associated with improved functional outcomes, including reduced drainage and no fistulotomy-related fecal incontinence, and resulted in wound healing in the majority of patients. These findings suggest that fistulotomy is a viable option and is not contraindicated in carefully selected patients with fistulizing pCD. However, given the risk of recurrence, caution should be exercised, and patients should receive a personalized, multidisciplinary approach, including both medical and surgical management, to achieve optimal fistula healing and low recurrence.

## Supplementary Information

Below is the link to the electronic supplementary material.

Supplementary file1 (DOCX 13 KB)

## Data Availability

The data used in this study are not publicly available but can be provided upon reasonable request.

## Declarations

### Originality

This study is original, written without the use of generative AI, has not been published elsewhere, and was presented at the American Society of Colon and Rectal Surgeons Annual Scientific Meeting on 1–4 June 2024, Baltimore, MD, USA.

### AI

During the preparation of this work, the author used PaperPal to proofread the grammar and punctuation. After using this tool/service, the author(s) reviewed and edited the content as needed and take full responsibility for the publication’s content.

## References

[1] Schwartz DA, Loftus EV, Tremaine WJ et al. (2002) The natural history of fistulizing Crohn’s disease in Olmsted County. Minnesota Gastroenterology 122:875–880 11910338 10.1053/gast.2002.32362

[2] Tsai L, McCurdy JD, Ma C et al. (2022) Epidemiology and natural history of perianal Crohn’s disease: a systematic review and meta-analysis of population-based cohorts. Inflamm Bowel Dis 28:1477–1484 34792604 10.1093/ibd/izab287 PMC9527611

[3] Geldof J, Iqbal N, LeBlanc JF et al. (2022) Classifying perianal fistulising Crohn’s disease: an expert consensus to guide decision-making in daily practice and clinical trials. Lancet Gastroenterol Hepatol 7:576–584 35325623 10.1016/S2468-1253(22)00007-3

[4] Wong S-Y, Rowan C, Diaz Brockmans E, et al. Perianal fistulizing Crohn’s disease-associated anorectal and fistula cancers: systematic review and expert consensus . Epub ahead of print 2024. 10.1016/j.cgh.2024.05.029. 10.1016/j.cgh.2024.05.029 38871152

[5] Lightner AL, Ashburn JH, Brar MS et al. (2020) Fistulizing Crohn’s disease. Curr Probl Surg 57:100808 33187597 10.1016/j.cpsurg.2020.100808

[6] Meima-van Praag EM, van Rijn KL, Wasmann KATGM, et al. (2022) Short-term anti-TNF therapy with surgical closure versus anti-TNF therapy in the treatment of perianal fistulas in Crohn’s disease (PISA-II): a patient preference randomised trial. *Lancet Gastroenterol Hepatol*. 7:617–626. 10.1016/S2468-1253(22)00088-7 35427495

[7] Wasmann KA, Joline de Groof E, Stellingwerf ME et al. (2020) Treatment of perianal fistulas in Crohn’s disease, seton versus anti-TNF versus surgical closure following anti-TNF [PISA]: a randomised controlled trial. J Crohn’s Colitis. 14:1049–1056 31919501 10.1093/ecco-jcc/jjaa004 PMC7476637

[8] Panes J, Reinisch W, Rupniewska E et al. (2018) Burden and outcomes for complex perianal fistulas in Crohn’s disease: systematic review. World J Gastroenterol 24:4821–4834 30479468 10.3748/wjg.v24.i42.4821 PMC6235801

[9] Vollebregt PF, van Bodegraven AA, Markus-de Kwaadsteniet TML et al. (2018) Impacts of perianal disease and faecal incontinence on quality of life and employment in 1092 patients with inflammatory bowel disease. Aliment Pharmacol Ther 47:1253–1260 29520808 10.1111/apt.14599 PMC5947114

[10] Gecse KB, Bemelman W, Kamm MA et al. (2014) A global consensus on the classification, diagnosis and multidisciplinary treatment of perianal fistulising Crohn’s disease. Gut 63:1381–1392 24951257 10.1136/gutjnl-2013-306709

[11] Graf W, Andersson M, Åkerlund JE et al. (2016) Long-term outcome after surgery for Crohn’s anal fistula. Colorectal Dis 18:80–85 26338142 10.1111/codi.13106

[12] Sangwan YP, Schoetz DJ, Murray JJ et al. (1996) Perianal Crohn’s disease: results of local surgical treatment. Dis Colon Rectum 39:529–535 8620803 10.1007/BF02058706

[13] Park MY, Yoon YS, Kim HE et al. (2021) Surgical options for perianal fistula in patients with Crohn’s disease: a comparison of seton placement, fistulotomy, and stem cell therapy. Asian J Surg 44:1383–1388 33966965 10.1016/j.asjsur.2021.03.013

[14] Herissay A, Siproudhis L, Balc’h E, et al. (2021) Combined strategies following surgical drainage for perianal fistulizing Crohn’s disease: failure rates and prognostic factors. Color Dis 23:159–168 10.1111/codi.15241 32640112

[15] Parks AG, Gordon PH, Hardcastle JD (1976) A classification of fistula‐in‐ano. Br J Surg 63:1–12 1267867 10.1002/bjs.1800630102

[16] Fichera A, Zoccali M (2015) Guidelines for the surgical treatment of Crohn’s perianal fistulas. Inflamm Bowel Dis 21:753–758 25738380 10.1097/MIB.0000000000000378

[17] Adamina M, Minozzi S, Warusavitarne J, et al. ECCO guidelines on therapeutics in Crohn’s disease: surgical treatment. 10.1093/ecco-jcc/jjae089. 10.1093/ecco-jcc/jjae089 38878002

[18] Gaertner WB, Burgess PL, Davids JS et al. (2022) The American Society of Colon and Rectal Surgeons clinical practice guidelines for the management of anorectal abscess, fistula-in-ano, and rectovaginal fistula. Dis Colon Rectum 65:964–985 35732009 10.1097/DCR.0000000000002473

[19] Papaconstantinou I, Kontis E, Koutoulidis V et al. (2017) Surgical management of fistula-in-ANO among patients with Crohn’s disease: analysis of outcomes after fistulotomy or seton placement—Single-center experience. Scand J Surg 106:211–215 27550245 10.1177/1457496916665763

[20] Shehab M, Alrashed F, Heron V et al. (2023) Comparative efficacy of biologic therapies for inducing response and remission in fistulizing Crohn’s disease: systematic review and network meta-analysis of randomized controlled trials. Inflamm Bowel Dis 29:367–375 35604382 10.1093/ibd/izac103

[21] Cohen BL, Lincango E, Holubar SD (2023) How to manage targeted immune suppressants (biologics and oral small-molecule drugs) perioperatively for inflammatory bowel disease and non-inflammatory bowel disease surgery. Clin Gastroenterol Hepatol 21:1148-1151.e1 36934049 10.1016/j.cgh.2023.01.042

[22] Laland M, François M, D’Amico F et al. (2023) Identification of the optimal medical and surgical management for patients with perianal fistulising Crohn’s disease. Color Dis 25:75–82 10.1111/codi.16314 36016511

[23] Cohen BL, Fleshner P, Kane SV et al. (2022) Prospective cohort study to investigate the safety of preoperative tumor necrosis factor inhibitor exposure in patients with inflammatory bowel disease undergoing intra-abdominal surgery. Gastroenterology 163:204–221 35413359 10.1053/j.gastro.2022.03.057

[24] Moosvi Z, Duong JT, Bechtold ML et al. (2021) Systematic review and meta-analysis: preoperative Vedolizumab and postoperative complications in patients with IBD. South Med J 114:98–105 33537791 10.14423/SMJ.0000000000001214

[25] Bachour SP, Shah RS, Joseph A et al. (2023) Change in biologic class promotes endoscopic remission following endoscopic postoperative Crohn’s disease recurrence. J Clin Gastroenterol. 10.1097/mcg.0000000000001943 10.1097/MCG.0000000000001943 PMC11128535 38019054

[26] Van Rijn KL, Meima-Van Praag EM, Bossuyt PM et al. (2022) Fibrosis and MAGNIFI-CD activity index at magnetic resonance imaging to predict treatment outcome in perianal fistulizing Crohn’s disease patients. J Crohn’s Colitis 16:708–716 34644395 10.1093/ecco-jcc/jjab168 PMC9228904

[27] Richard N, Derinck A, Bridoux V et al. (2024) Which magnetic resonance imaging feature is associated with treatment response in perianal fistulizing Crohn’s disease? Abdom Radiol. 10.1007/s00261-024-04238-3 10.1007/s00261-024-04238-3 38512515

[28] Crowley E, Ma C, Guizzetti L et al. (2024) Recommendations for standardizing MRI-based evaluation of perianal fistulizing disease activity in pediatric Crohn’s disease clinical trials. Inflamm Bowel Dis 30:357–369 37524088 10.1093/ibd/izad134

[29] Vuyyuru SK, Solitano V, Singh S et al. (2023) Scoring indices for perianal fistulising Crohn’s disease: a systematic review. J Crohns Colitis. 10.1093/ecco-jcc/jjad214 38126903 10.1093/ecco-jcc/jjad214

[30] Geldof J, Iqbal N, Warusavitarne J et al. (2022) The essential role of a multidisciplinary approach in inflammatory bowel diseases: the essential role of a multidisciplinary approach in inflammatory bowel diseases: combined medical-surgical treatment in complex perianal fistulas in CD. Clin Colon Rectal Surg 35:21 35069027 10.1055/s-0041-1740035 PMC8763455

[31] Kotze PG, Shen B, Lightner A et al. (2018) Modern management of perianal fistulas in Crohn’s disease: future directions. Gut 67:1181–1194 29331943 10.1136/gutjnl-2017-314918

