# Supplementary material for: Fistulotomy for superficial or minimal sphincter-involving fistulae in perianal Crohn’s disease: do they heal?
Source: Tech Coloproctol. 2026 May 13;30(1):99. doi: 10.1007/s10151-026-03351-3 (PMC13341898; doi:10.1007/s10151-026-03351-3)
Supplement: Supplementary file 1 — Supplementary file1 (DOCX 13 KB) [file 10151_2026_3351_MOESM1_ESM.docx]

**Supplemental Table.** Characteristics and complications of the second fistulotomy (N=43).

| **Variable** | **n=12** |
| --- | --- |
| **Interval 1^st^ to 2^nd^ Fistulotomy, months** | 9.2 (2.6, 19.0) |
| **2^nd^ Fistulotomy to last follow up, months** | 23.1 (7.2, 38.2) |
| **Second Fistulotomy**  Superficial/subcutaneous  Low fistulotomy (any muscle divided) | 9 (75%)  3 (25%) |
| **Complications**  No symptoms  Non-healing wounds  Pain  Mucopurulent drainage  Incontinence | 9 (75.0%)  1 (8.3%)  1 (8.3%)  1 (8.3%)  0 (0.0%) |

Data are presented as frequency (proportion) or median (IQR).
